# Complete Genome Sequence of Dengue Virus Serotype 2, Asian/American Genotype, Isolated from the Urine of a Venezuelan Child with Hemorrhagic Fever in 2016

**DOI:** 10.1128/genomeA.00529-18

**Published:** 2018-06-14

**Authors:** Gabriela M. Blohm, Alberto E. Paniz-Mondolfi, Marilianna C. Márquez, Julia C. Loeb, Carlos Pacheco, John A. Lednicky, Juliet R. C. Pulliam, J. Glenn Morris

**Affiliations:** a Department of Environmental and Global Health, College of Public Health and Health Professions, University of Florida, Gainesville, Florida, USA; b Emerging Pathogens Institute, University of Florida, Gainesville, Florida, USA; c The Venezuelan Science Research Incubator, Zoonoses and Emerging Pathogens Collaborative Network, Barquisimeto, Lara, Venezuela; d Department of Infectious Diseases and Tropical Medicine, IDB Biomedical Research Institute/IDB Clinic, Cabudare, Lara, Venezuela; e Directorate of Health, Department of Research and Academic Affairs, Instituto Venezolano de los Seguros Sociales (IVSS), Caracas, Venezuela; f Health Sciences Department, College of Medicine, Universidad Centroccidental Lisandro Alvarado, Barquisimeto, Lara, Venezuela; g Department of Pediatrics, Policlinica Barquisimeto, Barquisimeto, Lara, Venezuela; h DST-NRF Centre of Excellence in Epidemiological Modelling and Analysis (SACEMA), Stellenbosch University, Stellenbosch, South Africa; i Department of Medicine, College of Medicine, University of Florida, Gainesville, Florida, USA

## Abstract

The complete genome sequence was obtained for a Dengue virus 2 isolate from the urine of an 8-year-old girl who was hospitalized with dengue hemorrhagic fever in 2016 in Venezuela.

## GENOME ANNOUNCEMENT

Dengue virus serotypes 1 to 4 (DENV-1 to DENV-4) are distributed throughout Central and South America and the Caribbean (1). Symptoms of infection with DENV range from inapparent infection to dengue hemorrhagic fever (DHF) and dengue shock syndrome (DSS) (see https://www.cdc.gov/dengue/symptoms/index.html). DENV-1 to DENV-4 cocirculate in Venezuela (2), with the Asian/American (AS/AM) genotype of DENV-2 being associated with an increased incidence of DHF (3, 4). The AS/AM genotype of DENV-2 has been present throughout South America since the late 1970s (5). Several genetic subtypes of DENV-2 have been identified in Venezuela, suggesting that the virus has continued to evolve (6).

In April 2016, an 8-year-old girl presented to the hospital with a 2-day history of generalized petechiae, ecchymoses with acute onset, rectal bleeding, and occasional epistaxis. A week earlier, she had developed high-grade fever (38.5 to 39°C) followed by chills lasting 48 h, followed 3 days later by the appearance of a pruritic maculopapular rash. On admission, she was thrombocytopenic (platelet count, 25,000/μl), with a total leukocyte count of 9,200/μl, an elevated serum lactate dehydrogenase (LDH) level (443 IU/liter), and normal prothrombin time. She was conscious and well hydrated with stable blood pressure. In the hospital, there was continued spread of the ecchymoses, including ecchymotic skin lesions at venipuncture sites, and additional epistaxis, which prompted platelet transfusions. Hydration status was maintained, with no evidence of development of a shock syndrome. Her symptoms gradually resolved, with persistence of a few ecchymotic lesions that faded, and resolved uneventfully after 4 weeks.

Samples of blood and urine were collected 5 days after hospitalization. Serological tests revealed that she was positive for DENV IgG. Cryopreserved aliquots of the patient’s plasma and urine were shipped on dry ice to the University of Florida (UF), where the urine but not plasma tested positive for DENV-2 genomic RNA by reverse transcriptase-PCR (RT-PCR) (7). For verification purposes and to produce sufficient virus for sequence analyses, both plasma and urine were inoculated into LLC-MK2 (CCL-7), MRC-5, and Vero E6 (CRL-1586) cells, with mock-infected cells were kept in parallel. Cells inoculated with urine developed cytopathic effects (CPEs) 14 days postinoculation or later. No CPEs were formed in cells inoculated with plasma. Viral genomic RNA (vgRNA) was extracted from virions in spent cell growth medium using a QIAamp viral RNA minikit (Qiagen, Inc., Valencia, CA), and DENV-2 vgRNA was detected by reverse transcription quantitative PCR (qRT-PCR) (7). Dengue virus 2 vgRNA from the Vero cells was Sanger sequenced as previously described (8, 9), and the complete virus genome sequence was obtained and designated DENV-2 strain Homo sapiens/VEN-HUPAZ-1/2016.

The genome sequence of DENV-2 strain Homo sapiens/VEN-HUPAZ-1/2016 clusters within the AS/AM genotype and has high identity (99%) with various DENV-2 sequences obtained from viruses in Venezuela in 2007 (e.g., GenBank accession numbers HQ332185, HQ332190, and HQ332187). These genomes fall within Venezuelan AS/AM subcluster B (10).

### Accession number(s).

The complete genome sequence of DENV-2 strain Homo sapiens/VEN-HUPAZ-1/2016 has been deposited in the GenBank database under the accession number MH215277.
